# A Rare Case Report of Delayed Diagnosis of Superior Mesenteric Artery (SMA) Thrombosis Leading to Extensive Bowel Resection

**DOI:** 10.7759/cureus.86771

**Published:** 2025-06-25

**Authors:** Seshaan KNB, Ganesh Guru, T Raghupathy, Sairam KR, Keerthy Rajan

**Affiliations:** 1 General Surgery, Sree Balaji Medical College and Hospital, Chennai, IND

**Keywords:** delayed diagnosis, emergency, massive small bowel resection, sma thrombosis, smoking

## Abstract

A 60-year-old male presented with sudden-onset right-sided abdominal pain radiating to the back, along with vomiting and constipation for four days. Clinical examination revealed a distended and diffusely tender abdomen with absent bowel sounds. Contrast-enhanced computed tomography (CECT) of the abdomen showed near-complete thrombotic occlusion of the proximal superior mesenteric artery with distal reformation, infarcts in the liver and spleen, and features suggestive of small bowel necrosis and perforation. An emergency laparotomy revealed gangrene involving the distal jejunum, ileum, cecum, appendix, ascending colon, and part of the transverse colon. The patient underwent right hemicolectomy with resection of the distal jejunum and ileum, followed by distal jejunostomy and transverse colostomy. Histopathology confirmed transmural gangrene and thrombi in mesenteric vessels. This case describes the clinical presentation, radiological findings, surgical management, and histopathological features in a patient with extensive bowel involvement due to superior mesenteric artery thrombosis.

## Introduction

Acute mesenteric ischemia (AMI) is a surgical emergency resulting from reduced blood flow to the intestines, leading to ischemia and potentially irreversible bowel necrosis [1]. It accounts for approximately 1-2% of all acute abdomen cases but has a mortality rate of over 60% when the diagnosis is delayed. Among the various causes of AMI, superior mesenteric artery (SMA) thrombosis is particularly devastating due to its large vascular territory encompassing the midgut [2]. Prompt diagnosis remains a challenge due to nonspecific symptoms and limited early radiologic signs. This case emphasizes the importance of early recognition of vascular occlusion to prevent life-threatening complications and extensive bowel loss [3].

## Case presentation

A 60-year-old male presented to the emergency department with a two-day history of sudden-onset right-sided abdominal pain that radiated to the back. The pain was associated with multiple episodes of vomiting containing food particles but not bile or blood. He also reported constipation for the past four days, low-grade fever, and a significant loss of appetite. On general examination, he appeared acutely ill with a blood pressure of 140/90 mmHg and a pulse rate of 120 beats per minute. Abdominal examination revealed marked distension, generalized tenderness, tympanic note on percussion, and absent bowel sounds, all suggestive of intestinal obstruction or peritonitis. Baseline investigations, including complete blood count, serum electrolytes, renal parameters, and viral markers, were obtained, as shown in Table 1.

**Table 1:** Laboratory values at presentation

Parameter	Patient Value	Reference Range
Hemoglobin (Hb)	10.5 g/dL	13.0-17.0 g/dL
Total leukocyte count	18,200 /mm³	4,000-11,000 /mm³
Platelet count	310,000 /mm³	150,000-450,000 /mm³
Blood urea	52 mg/dL	10-40 mg/dL
Serum creatinine	1.5 mg/dL	0.6-1.3 mg/dL
Serum sodium	132 mEq/L	135-145 mEq/L
Serum potassium	4.3 mEq/L	3.5-5.0 mEq/L
Serum bicarbonate (HCO₃^-^)	18 mEq/L	22-28 mEq/L
Random blood sugar	168 mg/dL	70-140 mg/dL (non-fasting)
C-reactive protein (CRP)	88 mg/L	<5 mg/L
Serum lactate	5.2 mmol/L	0.5-2.2 mmol/L
Prothrombin time (PT)	17.8 seconds	11-13.5 seconds
International normalized ratio (INR)	1.4	0.8-1.2
Prothrombin concentration (PC)	58%	70-120%
D-dimer	3.1 µg/mL	<0.5 µg/mL

**Figure 1:**
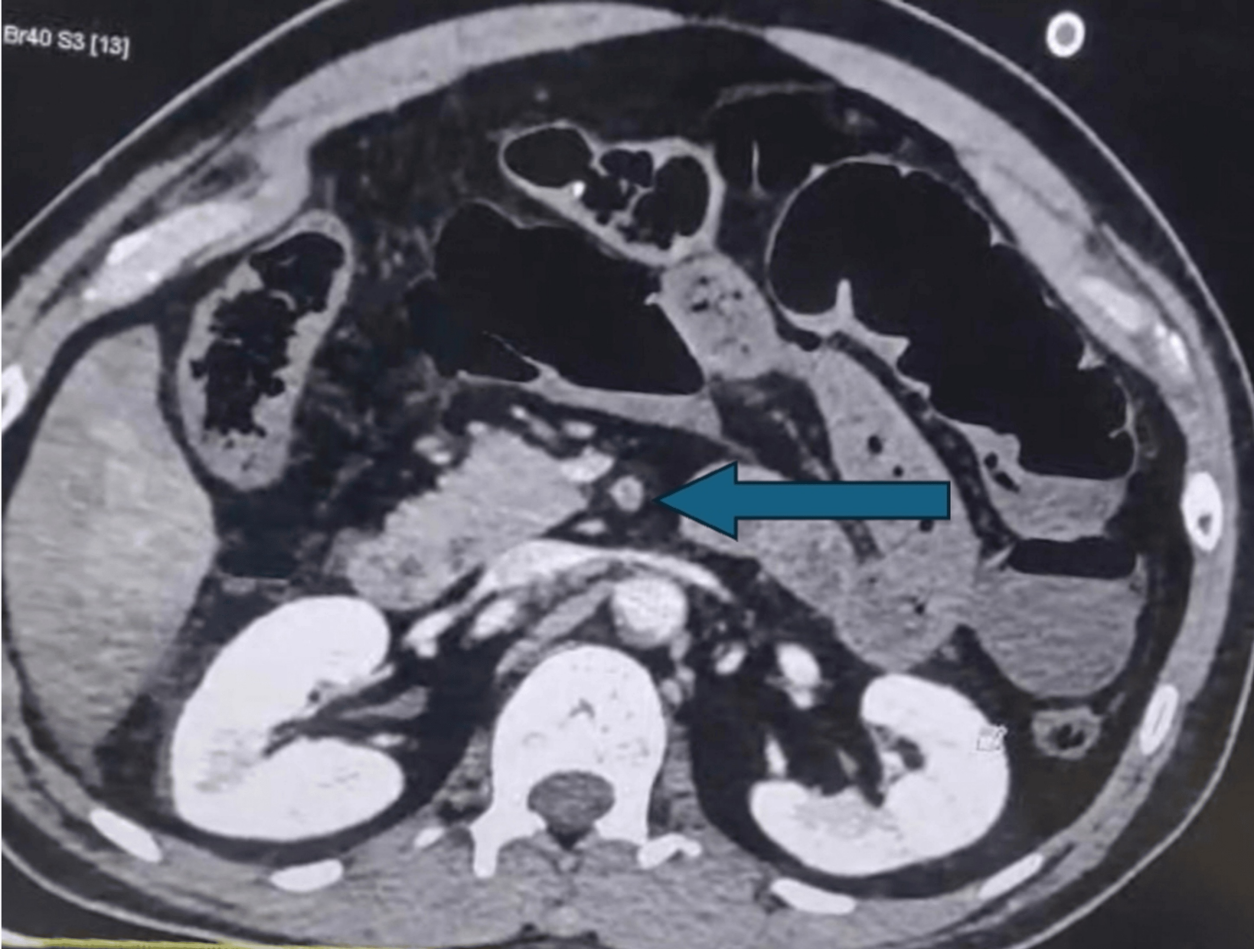
Axial contrast-enhanced computed tomography (CECT) image revealing proximal superior mesenteric artery (SMA) thrombosis The blue arrow points to a hypodense luminal filling defect within the proximal superior mesenteric artery, indicating acute thrombotic occlusion. Adjacent vascular segments appear narrowed, supporting the diagnosis of diffuse mesenteric ischemia.

A contrast-enhanced computed tomography (CECT) scan of the abdomen revealed a near-complete luminal filling defect with hypodense thrombotic content in the proximal segment of the SMA, with partial reformation of flow in the distal branches. Additionally, there was a faint and severely narrowed flow in the inferior mesenteric artery and the left renal artery. The scan also showed multiple calcific atheromatous plaques in the abdominal aorta and its branches, predominantly at the infrarenal level near the L3-L4 vertebra, as shown in Figure 1.

**Figure 2:**
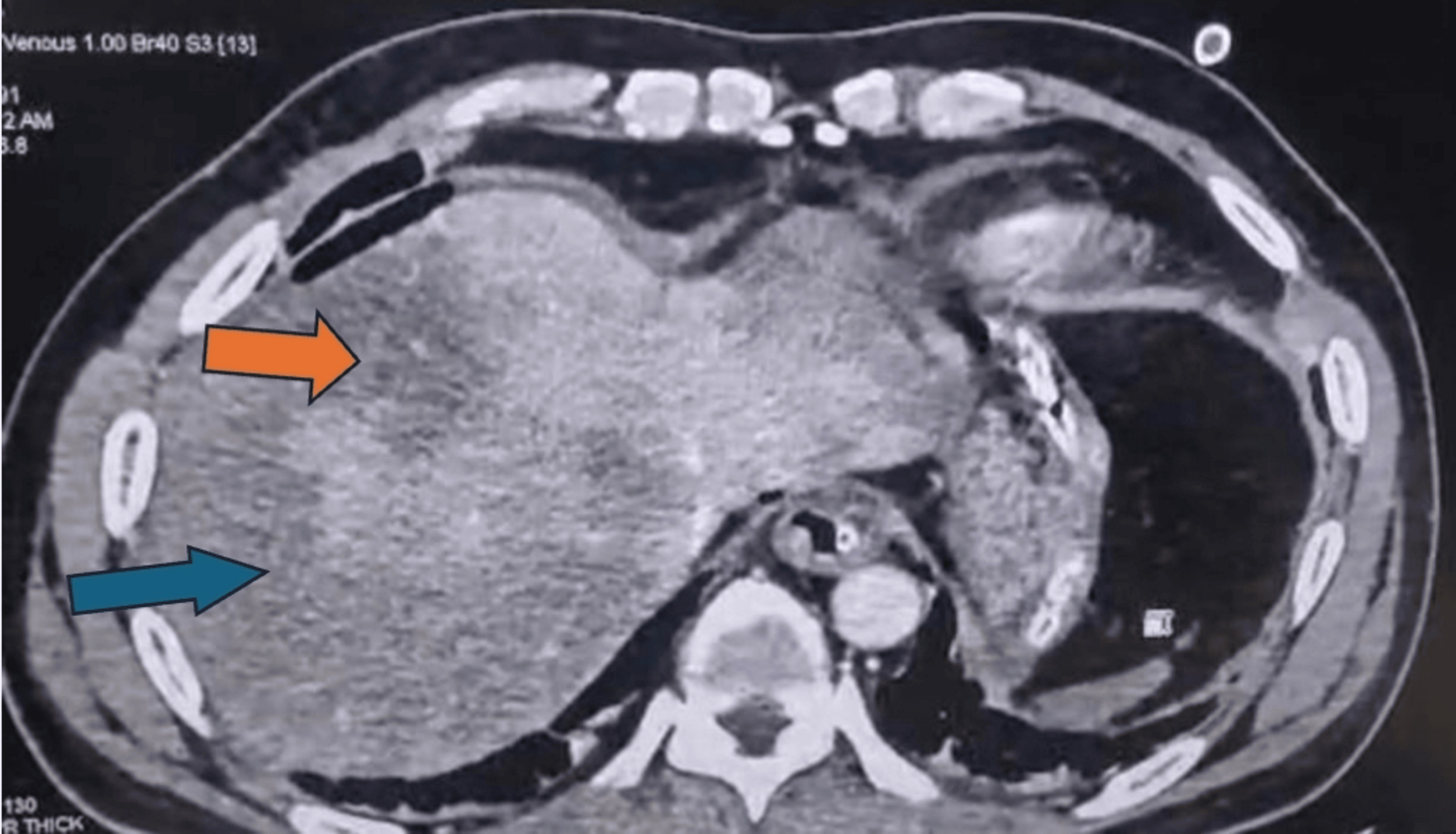
Axial contrast-enhanced computed tomography (CECT) image showing hepatic and splenic infarcts The orange arrow highlights diffuse hypoattenuation in the right lobe of the liver, while the blue arrow shows a non-enhancing, contracted spleen - both suggestive of infarcts secondary to systemic arterial thromboembolism.

Both hepatic lobes showed diffuse, non-enhancing hypodense areas, more prominent in the right lobe, consistent with infarction. The spleen was contracted and showed similar non-enhancing hypodense areas, also suggesting infarction, as shown in Figure 2.

**Figure 3:**
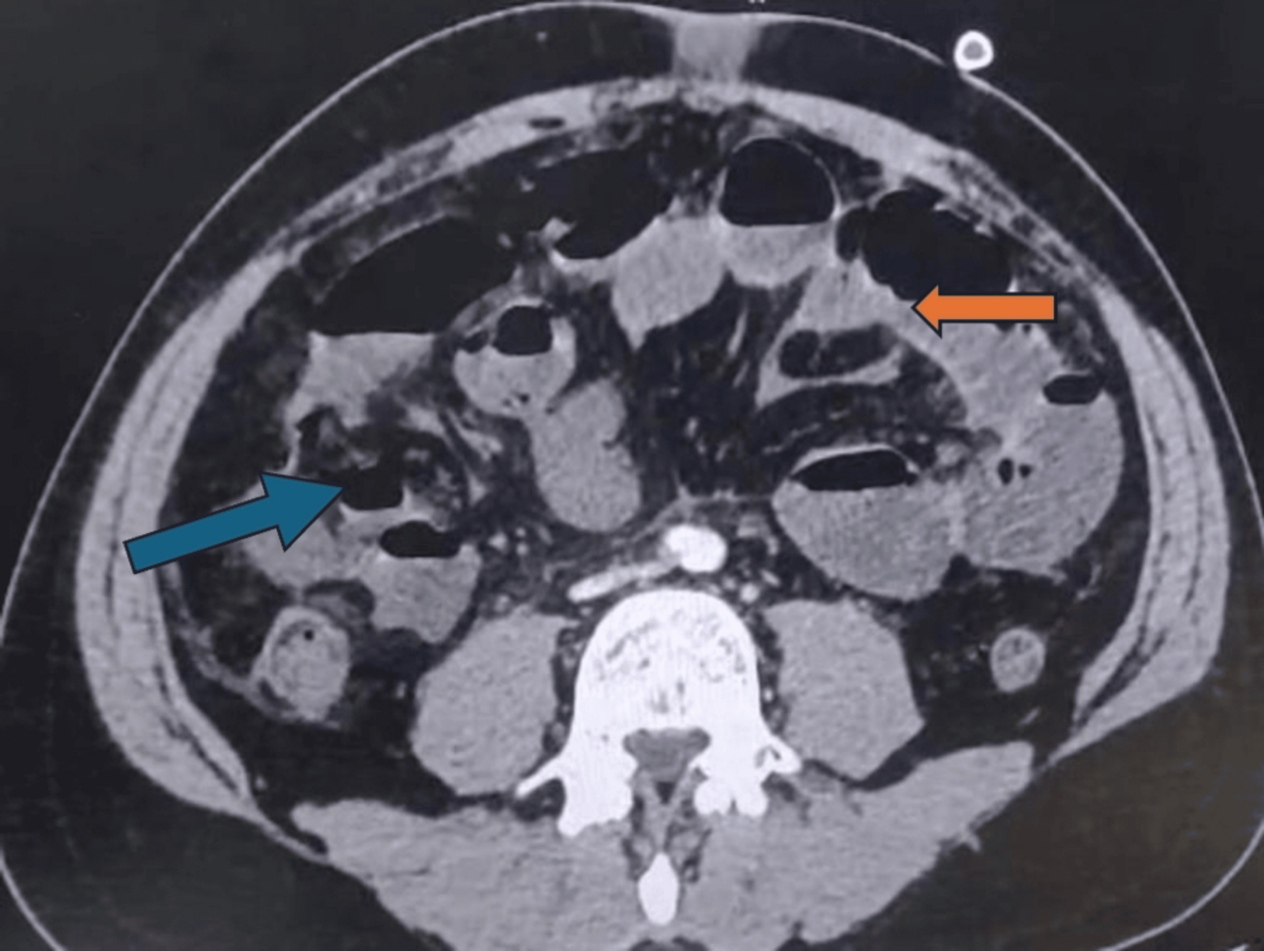
Axial contrast-enhanced computed tomography (CECT) image of the abdomen demonstrating bowel ischemia and pneumatosis The orange arrow indicates a segment of hypoenhancing bowel loop consistent with non-viable ischemic bowel. The blue arrow demonstrates intramural air within the bowel wall (pneumatosis intestinalis), indicative of transmural necrosis and impending perforation.

The small bowel loops were diffusely hypo-enhancing and dilated, especially the ileal segments, with evidence of multiple air-fluid levels. Several of these loops showed wall thinning and the presence of intramural air, suggestive of transmural necrosis. Overall, the imaging findings were suggestive of AMI secondary to arterial occlusion, as shown in Figure 3.

**Figure 4:**
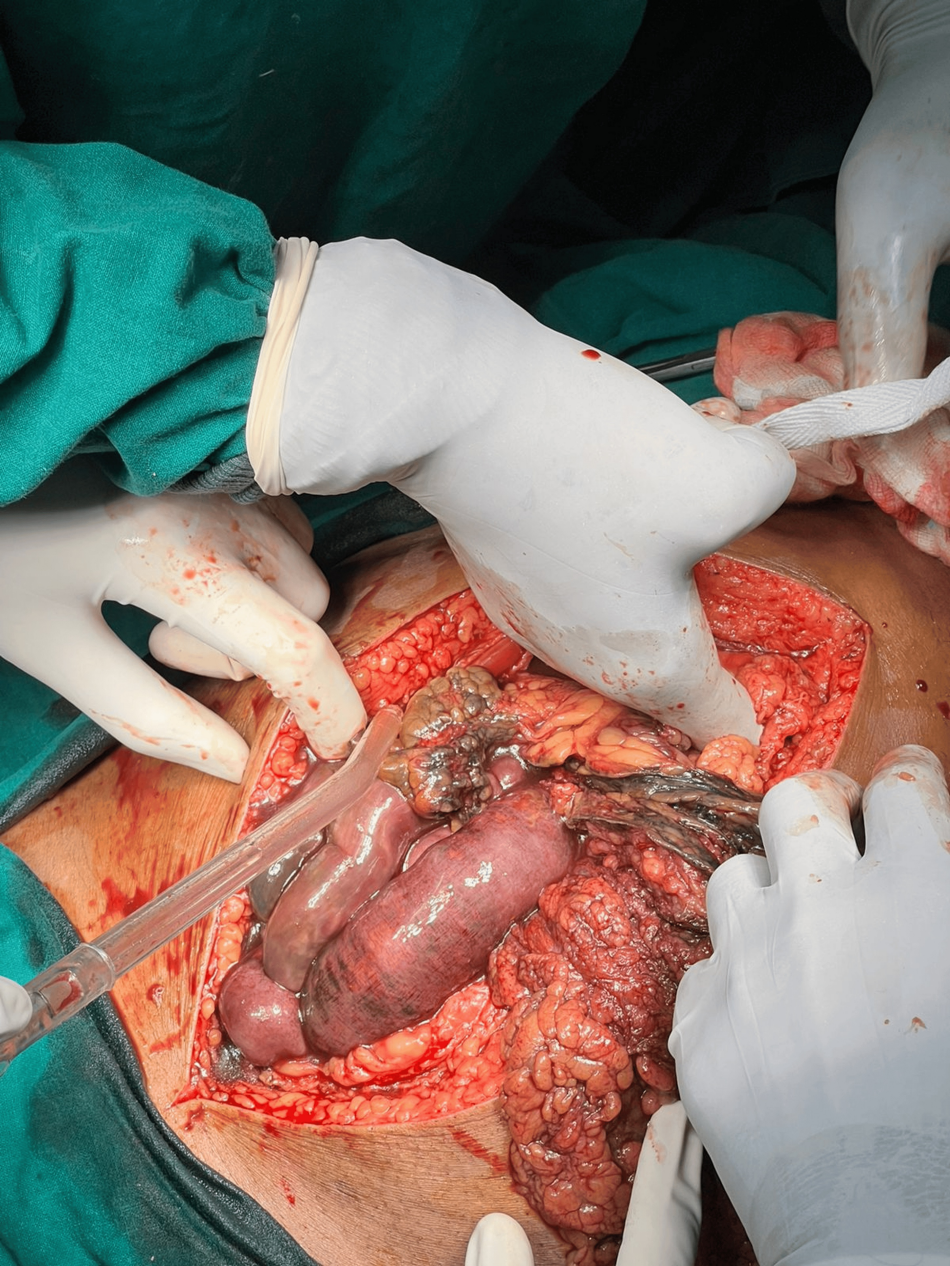
Intraoperative image showing dilated and gangrenous bowel loops A midline laparotomy revealing dusky, dilated, and thinned-out loops of distal jejunum, ileum, and large bowel consistent with extensive mesenteric ischemia secondary to superior mesenteric artery (SMA) thrombosis.

**Figure 5:**
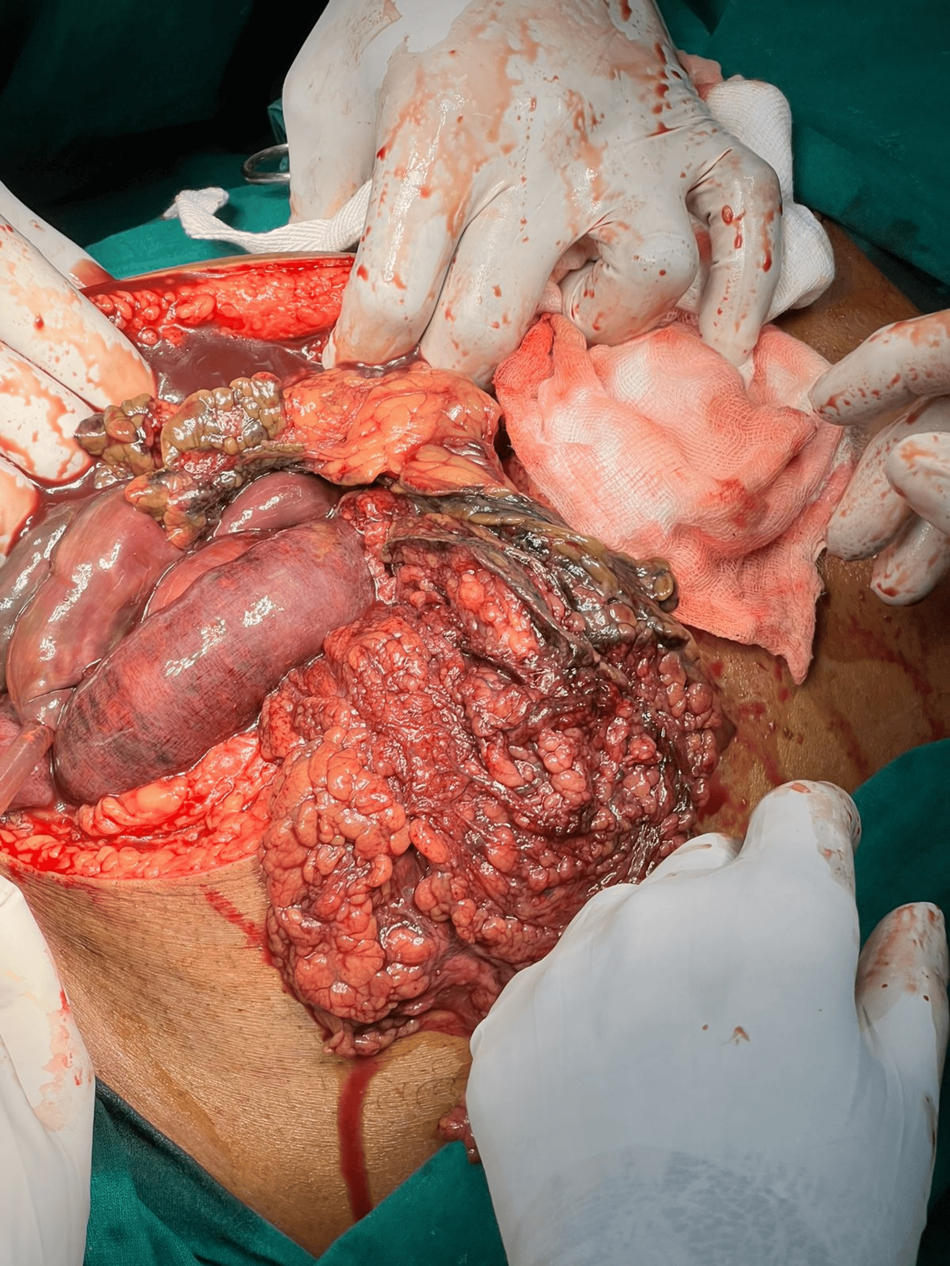
Further intraoperative exposure of gangrenous intestine View showing the extensive gangrenous involvement of the cecum, appendix, ascending colon, and part of the transverse colon.

The patient was resuscitated with intravenous fluids, broad-spectrum antibiotics, and analgesics and was taken up for emergency exploratory laparotomy. Upon opening the abdomen through a midline incision, extensive gangrene was identified involving the distal jejunum and ileum, along with the cecum, appendix, ascending colon, and one-third of the transverse colon (Figures 4-5). The ischemic bowel appeared dusky, thinned out, and non-viable, and the mesentery was noted to be thickened with visible thrombosed vessels. A right hemicolectomy with resection of the distal jejunum and ileum was performed. A distal jejunostomy and transverse colostomy were created.

**Figure 6:**
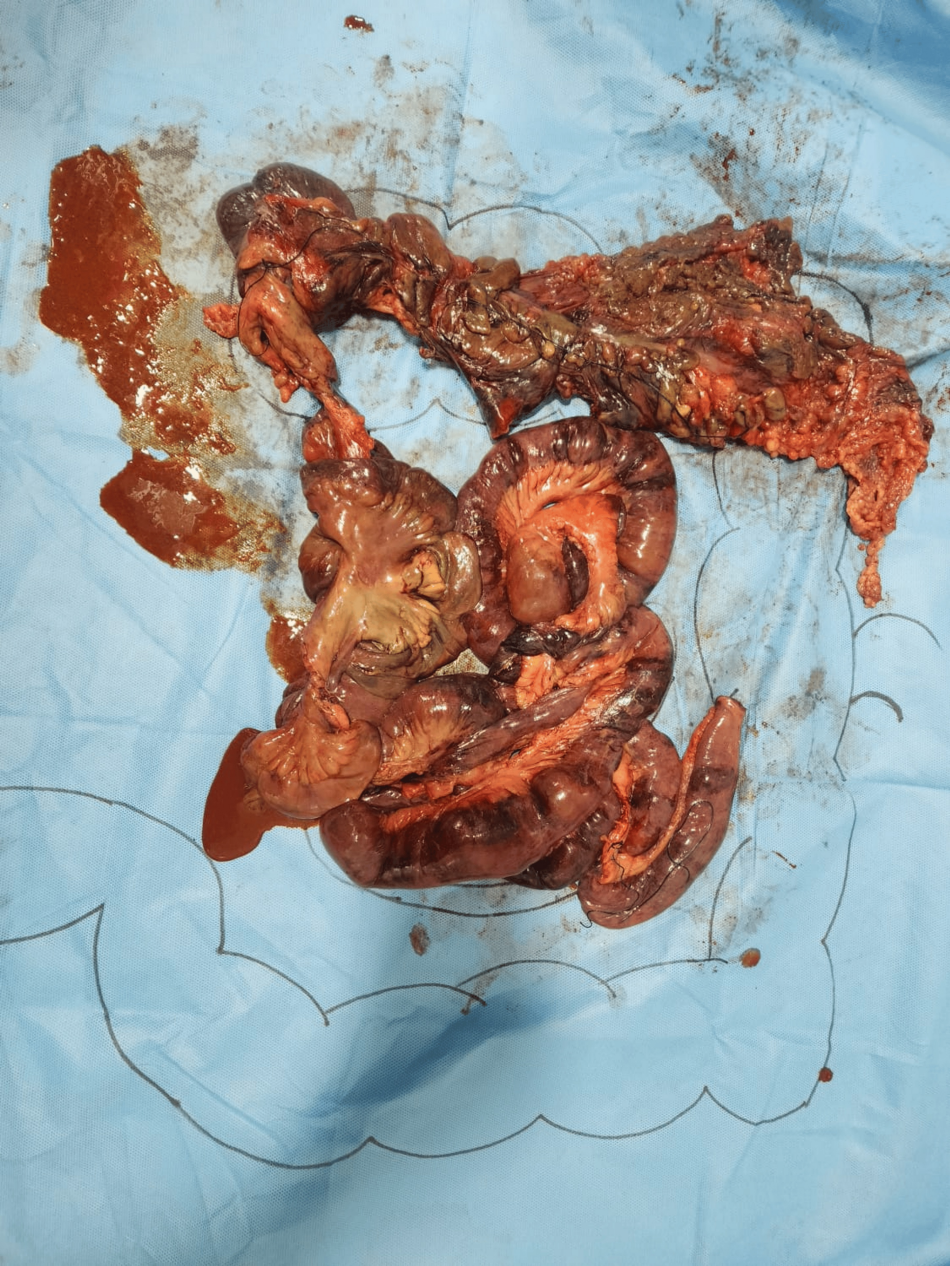
Gross resected specimen Specimen includes the distal jejunum, ileum, cecum with appendix, ascending colon, and part of transverse colon.

The resected specimen (Figure 6) measured approximately 243 cm in total, including 214 cm of small intestine (distal jejunum and ileum), 29 cm of large intestine (cecum, ascending colon, and a portion of the transverse colon), and the 4 cm long appendix. Gross examination revealed extensive gangrenous changes and multiple areas of wall thinning with black discoloration and perforations. The mesenteric vessels were dissected and found to be thrombosed. Histopathological analysis confirmed transmural necrosis of all intestinal layers in the distal jejunum, ileum, cecum, appendix, and colon. Fresh thrombi were identified in multiple mesenteric vessels, both arterial and venous, and one large artery showed features suggestive of pseudovasculitis. The proximal resected margin was noted to be only partially viable. These findings confirmed a diagnosis of extensive bowel infarction secondary to SMA thrombosis.

## Discussion

AMI is a life-threatening abdominal emergency resulting from compromised blood supply to the intestines. SMA thrombosis is an uncommon cause of AMI, but when present, it carries a high risk of bowel infarction and mortality. Although it represents only about 5-15% of all cases of AMI, SMA thrombosis, particularly due to atherosclerotic occlusion, typically occurs in older patients with predisposing vascular risk factors such as diabetes mellitus, hypertension, atrial fibrillation, and generalized atherosclerosis [1,2]. The patient in our case exhibited many of these risk features, including extensive calcific atheromatous disease noted on imaging. The classic presentation of AMI is the sudden onset of severe abdominal pain that is disproportionately greater than physical examination findings. As bowel infarction progresses, signs of peritonitis develop, as seen in our patient, who presented late with distension, diffuse tenderness, and absent bowel sounds. Nausea, vomiting, and signs of sepsis often follow, with systemic signs such as tachycardia and hypotension. Laboratory tests are generally nonspecific and may show leukocytosis, elevated lactate, or metabolic acidosis, but these markers do not establish the diagnosis definitively [3,4].

Contrast-enhanced computed tomography angiography (CTA) is considered the gold standard diagnostic modality in AMI. It allows rapid visualization of vascular occlusions, bowel viability, and associated complications such as infarction, perforation, or pneumatosis intestinalis. In this patient, CTA revealed near-complete thrombotic occlusion of the proximal SMA, partial reformation of distal flow, bowel wall thinning with intramural air, hepatic and splenic infarcts, and signs of bowel perforation. These findings strongly supported the diagnosis of arterial mesenteric ischemia with secondary necrosis [5,6]. SMA thrombosis usually occurs in a background of chronic mesenteric ischemia, superimposed by an acute event such as plaque rupture or thrombosis in situ. Our patient's extensive atherosclerotic burden seen on CT, along with calcific plaques in the abdominal aorta and iliac arteries, further supported the thrombotic mechanism. Chronic low-flow states or reduced collateralization due to arterial narrowing may predispose to catastrophic occlusion of the SMA trunk [7].

Histopathology in our case showed transmural necrosis of the jejunum, ileum, cecum, appendix, and proximal colon, confirming irreversible ischemia. The mesenteric vessels demonstrated fresh thrombi in both arteries and veins, and one large artery displayed histologic features of pseudovasculitis. Though true vasculitis is rare in AMI, similar histological patterns may occasionally be seen in ischemic vessels due to immune-mediated changes, especially in chronic ischemia [8,9]. Management of SMA thrombosis depends on the stage at diagnosis. Early-stage disease with no signs of infarction may be treated with anticoagulation or catheter-directed thrombolysis. However, once signs of bowel infarction, peritonitis, or perforation are evident, emergency laparotomy becomes mandatory [10,11]. Intraoperatively, bowel viability should be meticulously assessed, with resection of all necrotic segments and creation of stomas when required. In our case, over 240 cm of bowel (jejunum, ileum, colon) were non-viable and were resected. A distal jejunostomy and transverse colostomy were performed due to extensive involvement and hemodynamic instability. Re-anastomosis was deferred due to the high risk of leak in a septic setting.

Outcomes in SMA thrombosis depend heavily on timing. Delay in diagnosis beyond 24 hours is associated with a mortality rate exceeding 70-80%, primarily due to sepsis, short bowel syndrome, or multi-organ failure [12]. In our case, although extensive bowel resection was necessary, early surgical intervention following radiologic diagnosis likely contributed to survival. Long-term complications include nutritional deficiencies, stoma-related morbidity, and potential need for home parenteral nutrition in cases with short bowel syndrome [13].

Table 2 summarizes the key differentiating features of arterial and venous mesenteric ischemia.

**Table 2:** Comparison of arterial and venous mesenteric ischemia AF, atrial fibrillation; MI, myocardial infarction; SMA, superior mesenteric artery

Feature	Arterial Ischemia (SMA Thrombosis)	Venous Ischemia
Onset	Sudden, severe	Gradual, progressive
Pain	Out of proportion to the exam	Dull, colicky
Risk factors	Atherosclerosis, AF, MI	Hypercoagulability, malignancy
CT findings	Arterial occlusion, bowel hypoperfusion, pneumatosis	Venous filling defect, bowel wall thickening
Treatment	Surgical resection, revascularization	Anticoagulation, surgery if infarcted

Another important consideration is the extent of bowel resection required and the resulting residual length. Generally, at least 100 cm of small bowel with an intact colon is required to maintain enteral autonomy. In this case, although 214 cm of small bowel was resected, the remaining proximal jejunum and creation of a jejunostomy ensured functional preservation, though the patient may face nutritional challenges postoperatively [14].

In conclusion, early recognition of SMA thrombosis remains a significant clinical challenge. A high index of suspicion is vital in elderly patients with unexplained abdominal pain, especially with risk factors like atherosclerosis or atrial fibrillation. Timely radiological evaluation and prompt surgical intervention are essential to reduce morbidity and mortality associated with this devastating condition [15].

## Conclusions

This case demonstrated the complex clinical course of a patient with superior mesenteric artery thrombosis, who presented with delayed symptoms and signs of advanced bowel ischemia. The extensive involvement of both small and large bowel segments necessitated an emergency surgical intervention with major bowel resection, including a right hemicolectomy, distal jejunostomy, and transverse colostomy. Despite the severity of the ischemia and the extent of necrosis encountered intraoperatively, timely surgical decision-making based on radiological and clinical findings allowed for a controlled operative approach and patient stabilization. The case emphasizes how rapidly progressive ischemia can lead to catastrophic outcomes when presentation is delayed, and how surgical management must be adapted based on intraoperative findings. Careful attention to clinical deterioration and strategic use of imaging played a crucial role in this patient’s management. This experience underscores the importance of individualized clinical judgment and coordinated multidisciplinary care in managing advanced mesenteric ischemia.
